# A machine learning-based test for adult sleep apnoea screening at home using oximetry and airflow

**DOI:** 10.1038/s41598-020-62223-4

**Published:** 2020-03-24

**Authors:** Daniel Álvarez, Ana Cerezo-Hernández, Andrea Crespo, Gonzalo C. Gutiérrez-Tobal, Fernando Vaquerizo-Villar, Verónica Barroso-García, Fernando Moreno, C. Ainhoa Arroyo, Tomás Ruiz, Roberto Hornero, Félix del Campo

**Affiliations:** 1 Pneumology Department, Río Hortega University Hospital, Valladolid, Spain; 2 Biomedical Engineering Group, University of Valladolid, Valladolid, Spain; 3 Centro de Investigación Biomédica en Red en Bioingeniería, Biomateriales y Nanomedicina (CIBER-BBN), Valladolid, Spain

**Keywords:** Machine learning, Biomedical engineering

## Abstract

The most appropriate physiological signals to develop simplified as well as accurate screening tests for obstructive sleep apnoea (OSA) remain unknown. This study aimed at assessing whether joint analysis of at-home oximetry and airflow recordings by means of machine-learning algorithms leads to a significant diagnostic performance increase compared to single-channel approaches. Consecutive patients showing moderate-to-high clinical suspicion of OSA were involved. The apnoea-hypopnoea index (AHI) from unsupervised polysomnography was the gold standard. Oximetry and airflow from at-home polysomnography were parameterised by means of 38 time, frequency, and non-linear variables. Complementarity between both signals was exhaustively inspected via automated feature selection. Regression support vector machines were used to estimate the AHI from single-channel and dual-channel approaches. A total of 239 patients successfully completed at-home polysomnography. The optimum joint model reached 0.93 (95%CI 0.90–0.95) intra-class correlation coefficient between estimated and actual AHI. Overall performance of the dual-channel approach (kappa: 0.71; 4-class accuracy: 81.3%) significantly outperformed individual oximetry (kappa: 0.61; 4-class accuracy: 75.0%) and airflow (kappa: 0.42; 4-class accuracy: 61.5%). According to our findings, oximetry alone was able to reach notably high accuracy, particularly to confirm severe cases of the disease. Nevertheless, oximetry and airflow showed high complementarity leading to a remarkable performance increase compared to single-channel approaches. Consequently, their joint analysis via machine learning enables accurate abbreviated screening of OSA at home.

## Introduction

Recent epidemiological studies reported an increasing prevalence of obstructive sleep apnoea (OSA) among general population^1,2^, as well as a substantially greater prevalence in groups with particularly high risk for adverse consequences, such as patients with hypertension, cardiovascular disease, diabetes, or subjects evaluated for bariatric surgery^3^. Undiagnosed OSA is a major health burden worldwide due to the significant negative consequences for the patient^4^ and the increased utilisation costs for the healthcare system^5^. Therefore, timely and accurately diagnosis is essential for an appropriate management of the disease.

In order to increase availability and accessibility to diagnostic resources for early detection, unattended abbreviated testing based on the recording of a reduced number of physiological signals at home has being encouraged during the last years^6^. Despite well-known drawbacks such as higher risk of invalid study due to poor signal quality and inability to provide the actual total sleep time and to detect arousals, the American Academy of Sleep Medicine (AASM) recommends the use of abbreviated tests at home (type III and IV monitors) for initial screening of OSA under appropriate constrains^6^: uncomplicated adult patients showing symptoms indicative of high suspicion of moderate-to-severe OSA.

Despite exhaustive validation^7,8^, there is a great discrepancy on the use of type III monitors for extensive routine screening of sleep apnoea at home because set up complexity, time-consuming manual analysis, and intrusiveness for patients are still relevant. In this regard, type IV portable devices, characterised by the acquisition of just one or two channels, are expected to definitively overcome these drawbacks. Nonetheless, the most appropriate number and type of signals involving unsupervised monitoring remains unknown. Further research is needed to provide additional evidence on the most suitable way to maximise the performance of these simplified approaches.

In the present study, we focus on the usefulness of blood oxygen saturation (SpO_2_) and airflow, which are commonly involved in type IV devices. Individually, both signals have been found to provide relevant information for OSA diagnosis^9–12^. Notwithstanding, the potential complementarity of the features derived from both signals has been marginally studied^13^. SpO_2_ and airflow are both needed to score a hypopnoea event, which shows a relevant contribution to the overall apnoea-hypopnoea index (AHI) in several patients. Using either single-channel oximetry or airflow alone, we could lose essential information on the interaction between both signals, leading to important misdiagnosis. Therefore, we hypothesised that joint recording and analysis of SpO_2_ and airflow would be able to maximise diagnostic performance of abbreviated tests in the context of OSA screening. In this way, pattern recognition and machine-learning techniques have demonstrated unique usefulness in the characterisation of cardiorespiratory signals for automated OSA detection^14–18^. Particularly, support vector machines (SVMs) reached high diagnostic performance in binary classification problems (OSA-positive vs. OSA-negative) improving conventional approaches^15,19,20^. Despite being less used, SVMs have been adapted to accomplish regression analysis tasks as well^21^. As knowing the rate of respiratory events provides precise information on the actual severity status of a patient, we proposed to use regression SVMs to estimate the AHI from SpO_2_ and airflow, in order to thoroughly assess the contribution of each signal into a potential performance improvement.

Accordingly, this study is aimed at assessing whether joint analysis of SpO_2_ and airflow recordings by means of machine-learning algorithms leads to a significant diagnostic performance increase compared to single-channel approaches. In order to enhance generalisability of the research, all the sleep studies were carried out at home.

## Methods

### Population under study

Consecutive patients referred to the sleep unit of the Río Hortega University Hospital of Valladolid (Spain) were involved in the study. All patients showed moderate-to-high clinical suspicion of suffering from OSA due to at least one of the following symptoms: excessive daytime hypersomnolence, loud snoring, nocturnal choking and awakenings and/or witnessed apnoeas. Patients with a previous diagnosis and/or treatment for OSA, severe cardiovascular diseases, neuromuscular diseases, chronic respiratory failure or additional sleep disorders, such as narcolepsy, insomnia, periodic leg movements, restless legs syndrome, central sleep apnoea (>50% of total events categorised as central) or Cheyne-Stokes respiration, were excluded. Participants aged ≥18 years old. All were informed to participate in the study and signed an informed consent. The Ethics and Clinical Research Committee of the Río Hortega University Hospital (CEIC-HURH) approved the protocol of the study (approval number: CEIC 147/16), which was conducted according to the principles expressed in the Declaration of Helsinki.

G*Power 3.1 was used to estimate the sample size. Differences in mean and standard deviation among OSA severity degrees of relevant variables derived from oximetry and airflow were used to measure the effect size^15,17^. For a statistical power of 95% (significance level or type I error *α* = 0.05) a medium effect size equal to 0.45 was obtained, leading to a sample size of 252 patients. Considering a maximum rate of invalid unsupervised sleep studies equal to 20%, the estimated sample size for this research was 303 participants.

### Data collection protocol

Participants were asked for tobacco and alcohol consumption in order to characterise non-healthy habits. Clinical history was reviewed to confirm/discard the presence of frequent comorbidities, particularly chronic obstructive pulmonary disease, hypertension, and type 2 diabetes mellitus. Daytime somnolence was assessed by the Epworth Sleepiness Scale.

Unsupervised polysomnography (PSG) was carried out using an Embletta MPR with the ST + proxy (Embla Systems, Natus Medical Inc. CA, USA). Electroencephalogram (F3/C3/O1/F4/C4/O2), electrooculogram (left/right), chin electromyogram (left/right), tibial electromyogram (left/right), ECG, chest and abdominal movements by respiratory inductance plethysmography, airflow measured by both a nasal pressure transducer and an oral thermistor, position (triaxial accelerometer) and both SpO_2_ and pulse rate via pulseoximetry, were recorded at patients’ home. At-home sleep studies were programmed to start and finish automatically at 23:30 P.M. and 07:00 A.M., respectively (total recording time 450 min long). Trained nurses went to the patient’s home to attach sensors and set up the device. When all channels showed high signal quality (Embletta’s built-in quality measurement tool), nursing staff left the patient’s home. Next morning, the portable device was returned to the hospital, where a single trained expert downloaded the sleep study for subsequent offline analysis. Electroencephalographic and cardiorespiratory events were scored manually using AASM 2012 rules^22^. The AHI from portable PSG (AHI_PSG_) was used as gold standard to confirm OSA. All PSGs with a total sleep time <3 h due to bad signal quality (transient artefacts or sustained significant signal loss), premature battery depletion, or voluntary termination of the study by the patient, as well as those showing low sleep efficiency and/or no REM sleep, were withdrawn from the study.

### Automated analysis of oximetry and airflow

SpO_2_ and airflow were both obtained from unattended PSG at home and subsequently processed offline. SpO_2_ from nocturnal oximetry was recorded at a sampling rate of 75 Hz while the airflow signal from the nasal prong pressure was sampled at 250 Hz. According to the input signal, three expert systems for automated estimation of the AHI were designed and prospectively assessed: (1) single-channel SpO_2_, (2) single-channel airflow, and (3) dual-channel input composed of simultaneous SpO_2_ and airflow recordings. In every branch of the methodology, four common signal-processing stages were applied to maximise the diagnostic performance of the signal: pre-processing, feature extraction, dimensionality reduction, and pattern recognition. Automated dimensionality reduction and pattern recognition stages were performed using a training dataset for appropriate feature selection and optimisation of the AHI regression models, respectively. Finally, agreement and diagnostic performance of the three proposed models were assessed in an independent test dataset. A detailed flowchart showing the procedures and the datasets involved at each stage of the methodology can be found as Supplementary Fig. S1.

#### Pre-processing

SpO_2_ recordings were automatically pre-processed to remove oximetric samples under 50% and transient deeps commonly linked with patient’s movements. Next, all oximetry signals were downsampled to 3 Hz to accomplish feature extraction^22^. Regarding airflow recordings, firstly, segments showing sustained malfunctioning were removed. Then, a low-pass filter with a cut-off frequency of 1.2 Hz was applied to reduce noise^17^. All recordings, both SpO_2_ and airflow, with a total recording time <4 h after pre-processing were discarded due to insufficient data for accurate estimation of the AHI from a single/dual-channel approach^6^.

#### Feature extraction

SpO_2_ and airflow signals were parameterised both in the time and frequency domains. Statistical, spectral, and non-linear features, as well as conventional oximetric and respiratory disturbance indices commonly used in the context of automated OSA diagnosis were computed^14–17,23^.

- Statistics in the time domain. The widely known mean (*M1t*), variance (*M2t*), skewness (*M3t*), and kurtosis (*M4t*) were computed to quantify the position, width, asymmetry, and peakedness of the normalised data histogram of SpO_2_ and airflow amplitudes in the time domain.
- Measures in the frequency domain. The power spectral density (PSD) function of every SpO_2_ and airflow recording was computed to estimate the power spectrum of the signal. An OSA-related frequency band was defined for each kind of signal (SpO_2_ and airflow) based on previous studies: 0.014 to 0.033 Hz for oximetry^14^ and 0.025 to 0.050 Hz for airflow^17^. Then, the mean, variance, skewness, and kurtosis were derived from the histogram of spectral amplitudes (*M1f* to *M4f*). The Shannon spectral entropy (*SE*), the median frequency (*MF*), and the Wootters distance (*WD*), which have been previously found to provide essential OSA-related information from oximetry and airflow, were also computed^15,17^. Finally, amplitude- and power-based measures were computed to further characterise each spectral band of interest: maximum (*MA*) and minimum (*mA*) amplitudes as well as relative power (*PR*) were calculated.
- Non-linear features. Sample entropy (*SampEn*), central tendency measure (*CTM*), and Lempel-Ziv complexity (*LZC*) were applied to obtain non-linear measures of irregularity, variability, and complexity commonly present in biological systems^15,17^.
- Conventional oximetric and disturbance indices. Despite evidences showing an intrinsic underestimation^24^, conventional indices based on the number of oximetric and respiratory events and the severity of desaturations have been found to be very useful in OSA detection, particularly when they are used together with additional automated features^16,25^. Consequently, the commonly used oxygen desaturation index ≥3% (ODI3) and ≥4% (ODI4) and the respiratory disturbance index (RDI) from airflow, as well as the minimum (Sat_MIN_) and the average (Sat_AVG_) saturation values and the cumulative time spent with a saturation below 90% (CT90) were computed.

Finally, according to the data source, three initial feature sets were built: (1) single-channel SpO_2_ feature set, composed of 21 features from oximetry; (2) single-channel airflow feature set, composed of 17 features from airflow; and (3) dual-channel feature set, composed of 38 features derived from the combination of all the variables from SpO_2_ and airflow.

#### Dimensionality reduction

The fast correlation-based filter (FCBF) was applied for suitable feature selection owing the usefulness reported in previous studies in the context of OSA screening from oximetry^26^ and airflow^16,17^. FCBF is able to detect the most relevant as well as non-redundant variables governing a system^27^. Feature selection is accomplished based on the characteristics of the problem under study, e.g., the AHI of each patient. An optimum feature subset is obtained independently of the particular algorithm used for subsequent pattern recognition, thus allowing for high generalisability^27^. Additionally, in order to avoid dependence on a particular training dataset, a bootstrapping approach was implemented. Accordingly, FCBF was repeated using 1000 bootstrap replicates derived from the training set. The significance of each feature was defined as the number of times each input variable was selected. Finally, variables showing higher significance than the average relevance for the whole input feature set were selected.

#### Pattern recognition using support vector machines

SVMs are non-linear algorithms originally designed to perform binary classification tasks^21^. In this regard, SVMs have been previously applied to distinguish between OSA-positive and OSA-negative patients using input patterns from ECG^19,20^ or oximetry^15^ signals, reaching high diagnostic performance in both problems. In addition, based on the principles of statistical learning theory, they have been adapted to accomplish regression analysis tasks as well^28^. As under the most common classification approach, the learning stage of a SVM algorithm for regression is based on the principle of structural risk minimisation. This way, high performance is achieved on training data while avoiding overfitting, leading to high generalisation capability. Two user dependent parameters have to be tuned to maximise accuracy: a regularisation parameter (*C*), which governs the trade-off between performance and model complexity; and the width of the Gaussian (*sigma*) of a radial basis function (RBF) kernel function, which represents a transformed feature space where separation of patterns is maximal. In the present study, a leave-one-out cross-validation procedure is applied in the training dataset to properly adjust these parameters. The widely used values 10^−3^, 10^−2^, …, 10^3^, 10^4^ were assessed for the regularisation parameter *C*, whereas 10^−2^, 10^−1^, …, 10^2^, 10^3^ were used for *sigma*, with a more accurate search round 10^2^, where a local maxima was found. The intra-class correlation coefficient (ICC) between the AHI from at-home PSG and the estimated AHI was used to drive model selection. Once optimised, the final model was trained using the whole training population. Three regression models were composed: (1) SVM_SpO2_, which provides the estimated AHI from single-channel oximetry; (2) SVM_AF_, which provides the estimated AHI from single-channel airflow; and (3) SVM_SpO2+AF_, which provides the estimated AHI from the dual-channel input that combines features from oximetry and airflow. Then, these models were prospectively assessed in an independent test dataset.

### Statistical analysis

Matlab R2017a (The MathWorks Inc., Natick, Massachusetts) was used to implement signal processing and pattern recognition algorithms, as well as to perform statistical analyses. The median value and interquartile range were computed to perform a descriptive analysis of variables involved in the study. The population was divided into training (60% first consecutive patients) and test (40% remaining consecutive patients) datasets. Normal distribution of input features was assessed by means of the Kolmogorov–Smirnov’s test, whereas the Levene’s test was used to assess homogeneity of variances. Accordingly, the non-parametric Mann-Whitney U test was used to assess differences in socio-demographic, anthropometric, and clinical variables from these datasets. The Chi^2^ test was used for categorical variables. A *p*-value <0.01 was considered significant.

The ICC was computed to quantitatively measure the agreement between the actual AHI from unattended PSG and the estimated AHI from SVM models, while Bland-Altman and Mountain plots were used for qualitative analysis of agreement. Additionally, the four common severity groups of OSA were considered (No-OSA: AHI < 5 events/h; mild: 5 ≤ AHI < 15 events/h; moderate: 15 ≤ AHI < 30 events/h; severe: AHI ≥ 30 events/h) and both the kappa coefficient and the overall accuracy were computed from the 4-class confusion matrices of each model in the independent test set.

Finally, the diagnostic performance was assessed for common binary cut-offs for mild (AHI ≥ 5 events/h), moderate (AHI ≥ 15 events/h), and severe (AHI ≥ 30 events/h) OSA. The widely known pairs of metrics from the 2-class confusion matrices were computed in the test dataset: sensitivity (Se) *vs*. specificity (Sp), positive predictive value (PPV) *vs*. negative predictive value (NPV), and positive likelihood ratio (LR+) *vs*. negative likelihood ratio (LR−). In addition, the accuracy (Acc) and the area under the receiver operating characteristics curve (AUC) were computed as overall measures of diagnostic performance. The 95% confidence interval (95%CI) was computed for every metric using bootstrap. The recommendations of the STARD group for reporting diagnostic accuracy studies were considered^29^.

## Results

**Figure 1:**
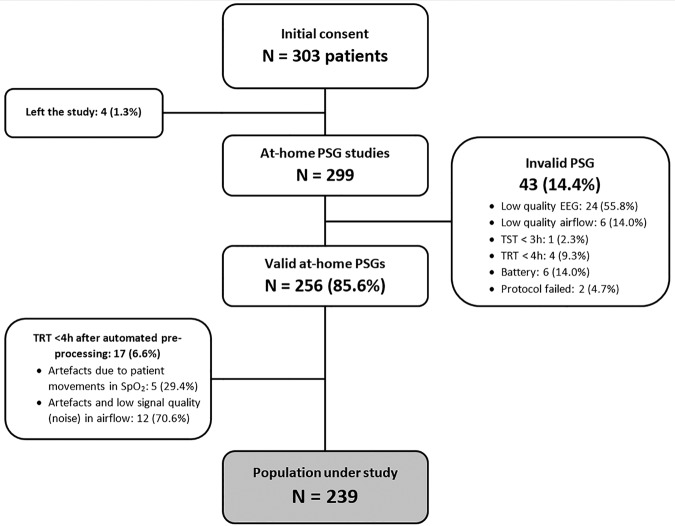
Patient recruitment flowchart. PSG: polysomnography; TRT: total recording time; TST: total sleep time.

A total of 303 eligible patients with suspicion of suffering from OSA were involved in the study from July 2016 to September 2017. Figure 1 shows the patient flowchart with a detailed description of the recruitment process. Regarding unattended PSG, 43 participants were withdrawn due to poor signal quality, while 17 patients were further removed during automated signal pre-processing. Finally, 239 patients successfully passed to the pattern recognition stage. Table 1 shows the main characteristics of the population under study. Polysomnographic variables from at-home PSG are summarised in Table 2.

**Table 1:** Main characteristics of the entire population under study and training and test groups.

	All	Training group	Test group	*p*-value
N° of subjects (n, %)	239	143	96	—
Age (years)	56.0 [46.0, 65.0]	55 [45.3, 64.0]	58.5 [48.5, 67.0]	0.157
N° of males (n, %)	164 (68.6%)	97 (67.8%)	67 (69.8%)	0.778
BMI (kg/m^2^)	28.4 [25.8, 32.4]	28.1 [25.5, 32.6]	28.8 [26.6, 32.1]	0.322
**Smoking status**				
Never-smoker	124 (51.9%)	72 (50.4%)	52 (54.2%)	0.782
Ex-smoker	86 (36.0%)	54 (37.8%)	32 (33.3%)	
Current smoker	29 (12.1%)	17 (11.9%)	12 (12.5%)	
**Alcohol consumption**	8 (3.3%)	7 (4.9%)	1 (1.0%)	0.104
**Daytime somnolence**				
ESS	11 [7, 15]	11 [7, 15]	10 [7, 14]	0.400
**Comorbidities**				
COPD	13 (5.4%)	6 (4.2%)	7 (7.3%)	0.301
HT	81 (33.9%)	43 (30.1)	38 (39.6%)	0.128
DM	28 (11.7%)	21 (14.7)	7 (7.3%)	0.081
**OSA severity**				
N° of patients AHI < 5 events/h	15 (6.3%)	9 (6.3%)	6 (6.3%)	0.999
N° of patients 5 ≤ AHI < 15 events/h	54 (22.6%)	38 (26.6%)	16 (16.7%)	0.084
N° of patients 15 ≤ AHI < 30 events/h	56 (23.4%)	29 (20.3%)	27 (28.1%)	0.165
N° of patients AHI ≥ 30 events/h	114 (47.7%)	67 (46.9%)	47 (49.0%)	0.792

Data are presented as median [interquartile range] or number (percentage). AHI: apnoea-hypopnoea index; BMI: body mass index; COPD: chronic obstructive pulmonary disease; DM: diabetes mellitus; ESS: Epworth sleepiness scale; HT: hypertension.

**Table 2:** Polysomnographic variables derived from unattended PSG at patient’s home.

	All (*N* = 239)	Training (*N* = 143)	Test (*N* = 96)	*p*-value
**Overall analysis of the recording**				
TRT (h)	450.0 [449.9, 450.0]	450.0 [450.0, 450.0]	450.0 [419.3, 450.0]	—
TST (h)	392 [348.8, 417.8]	395.5 [369.6, 423.2]	380.8 [326.5, 411.8]	<0.01
Sleep eff. (%)	89.1 [82.8, 93.9]	89.1 [82.8, 94.2]	89.5 [82.8, 92.7]	0.453
Sleep lat. (min)	7.5 [0.0, 24.8]	8.5 [0.0, 25.4]	5.5 [0.0. 23.7]	0.370
**Sleep staging**				
N1 (%)	11.6 [7.4, 18.0]	11.8 [6.8, 19.6]	11.6 [8.0, 16.3]	0.689
N2 (%)	35.6 [29.9, 43.7]	36.7 [30.9, 44.9]	34.1 [28.5, 40.6]	0.018
N3 (%)	27.7 [20.9, 34.2]	26.3 [19.7, 32.9]	30.0 [24.3, 36.2]	<0.01
REM (%)	22.6 [18.0, 26.1]	22.7 [18.1, 26.4]	22.5 [17.8, 25.8]	0.633
REM lat. (min)	69.0 [47.3, 105.0]	67.5 [46.1, 107.6]	71.8 [49.5, 101.0]	0.688
Total Ar (events/h)	20.2 [13.1, 31.6]	21.9 [13.6, 33.7]	18.0 [11.7, 28.1]	0.032
Resp. Ar (events/h)	11.8 [5.9, 21.3]	12.8 [6.1, 25.1]	10.7 [5.5, 16.7]	0.165
**Respiratory events**				
AHI (events/h)	27.2 [12.6, 45.6]	27.2 [11.4, 47.6]	26.2 [15.3, 44.4]	0.915
HI (events/h)	18.9 [9.2, 28.2]	17.1 [8.9, 26.3]	20.3 [11.9, 30.3]	0.093
AI (events/h)	5.0 [1.1, 15.7]	5.7 [1.4, 16.8]	4.1 [0.9, 12.6]	0.128
Obstructive/mixed events (%)	96.4 [89.7, 99.5]	95.8 [89.3, 99.2]	96.6 [91.7, 99.7]	0.199
Central events (%)	3.6 [0.6, 10.3]	4.2 [0.8, 10.7]	3.4 [0.3, 8.3]	0.199
Supine position (%)	39.9 [22.6, 59.5]	41.4 [27.6, 60.7]	33.4 [18.1, 58.3]	0.063
Events Avg time (s)	22.4 [20.2, 25.5]	22.2 [20.6, 25.1]	23.0 [19.9, 26.8]	0.279
Events Max time (s)	54.9 [44.0, 71.1]	55.0 [45.2, 67.3]	54.0 [43.2, 72.6]	0.627
**Oximetry**				
Sat Ini (%)	94.0 [92.9, 95.1]	93.7 [92.8, 95.0]	94.0 [92.8, 96.0]	0.410
Sat Avg (%)	92.5 [91.1, 94.0]	92.5 [91.1, 94.1]	92.6 [91.1, 93.9]	0.763
Sat Min (%)	83.0 [77.0, 87.0]	83.0 [76.3, 87.0]	83.0 [77.0, 86.0]	0.775
CT90 (%)	4.4 [0.4, 17.9]	4.2 [0.3, 17.6]	4.7 [0.6, 20.9]	0.791
ODI3 (events/h)	22.4 [11.1, 45.8]	25.1 [10.7, 46.2]	21.9 [12.1, 42.9]	0.937

Data are presented as median [interquartile range]. AI: apnoea index; AHI: apnoea-hypopnoea index; CT90: cumulative time spent with a saturation below 90%; Events Avg time: average duration of events; Events Max time: maximum duration of events; HI: hypopnoea index; N1: percentage of sleep time in N1 stage; N2: percentage of sleep time in N2 stage; N3: percentage of sleep time in N3 stage; ODI3: number of desaturations ≥3% per hour of sleep; REM: percentage of sleep time in rapid eye movement sleep; REM lat: REM stage latency; Resp Ar: respiratory arousal index; Sat Avg: average saturation; Sat Ini: initial saturation; Sat Min: minimum saturation; Sleep eff: sleep efficiency; Sleep lat: sleep latency; Total Ar: total arousal index; TRT: total recording time; TST: total sleep time.

**Figure 2:**
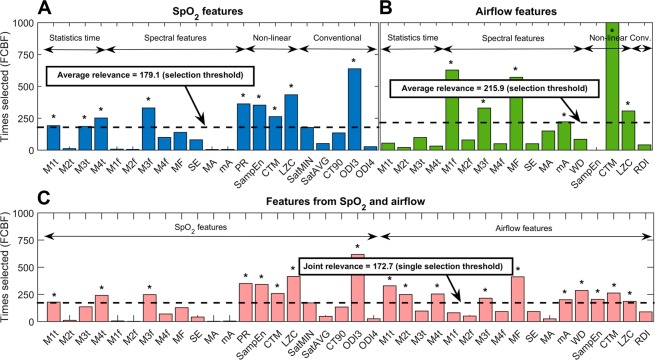
Automated feature selection procedure using a FCBF-based bootstrap (1000 iterations) approach for the proposed data sources: (**A**) single-channel oximetry; (**B**) single-channel airflow; and (**C**) dual-channel SpO_2_ and airflow. In the upper panels, variables are grouped according to the signal processing methodology: statistics in the time domain, spectral features, non-linear measures, and conventional indices. In the lower panel, variables are presented in the same order. For each data source, the particular significance threshold for feature selection is plotted (dashed black line). Selected optimum variables with relevance above the threshold are marked with an asterisk. M1t-M4t: 1^st^ to 4^th^ order statistical moments in the time domain; M1f-M4f: 1^st^ to 4^th^ order statistical moments in the apnoea-related frequency band; SE: Shannon spectral entropy; MF: median frequency; WD: Wootters distance; MA: maximum amplitude in the spectral band; mA: minimum amplitude in the spectral band; PR: relative power; SampEn: sample entropy; CTM: central tendency measure; LZC: Lempel-Ziv complexity; ODI3: oxygen desaturation index of 3%; ODI4: oxygen desaturation index of 4%; Sat_MIN_: minimum saturation; Sat_AVG_: average saturation; CT90: cumulative time spent with a saturation below 90%; RDI: respiratory disturbance index.

Figure 2 shows the feature selection process for the proposed data sources. From an initial feature set composed of 21 variables from single-channel SpO_2_, 9 (42.9%) optimum features were selected. Similarly, 6 out of 17 (35.3%) optimum features from single-channel airflow were automatically selected. Finally, a total of 18 out of 38 (47.4%) variables composed the optimum feature subset when both oximetry and airflow are considered jointly (dual-channel approach).

Regarding the optimisation process of each model during the training stage, SVM_SpO2_ maximises ICC for *C* = 10^4^ and *sigma* = 250 (maximum ICC_training_ = 0.94 from leave-one-out cross-validation), SVM_AF_ for *C* = 10^4^ and *sigma* = 20 (maximum ICC_training_ = 0.86), and SVM_SpO2+AF_ for *C* = 10^4^ and *sigma* = 100 (maximum ICC_training_ = 0.96). Supplementary Fig. S2 shows the optimisation process of the SVM input-parameters *C* and *sigma* for each model.

**Figure 3:**
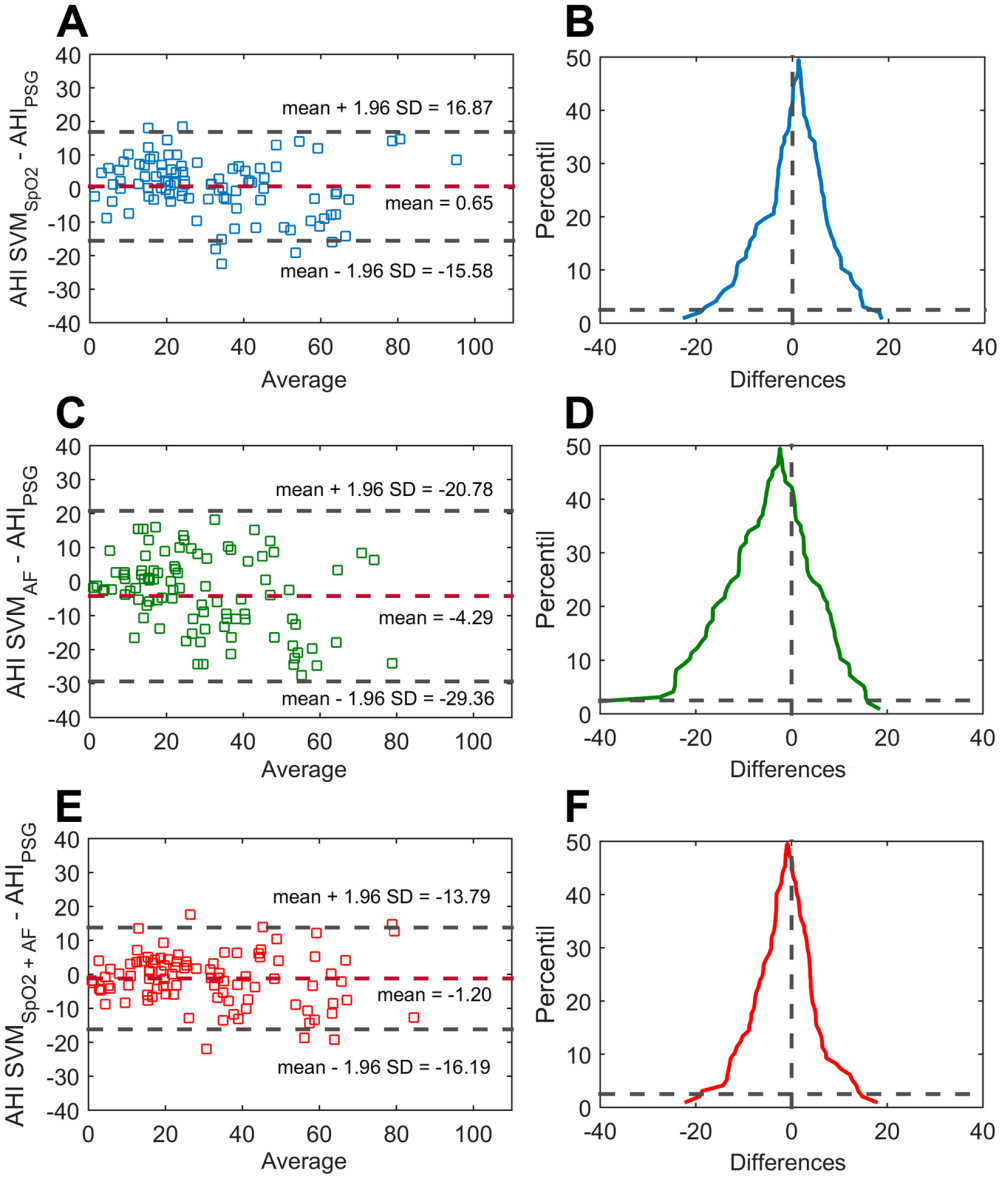
Bland-Altman and Mountain plots for characterising agreement between actual AHI from PSG and the estimated AHI derived from (**A**,**B**) single-channel SpO_2_, (**C**,**D**) single-channel airflow, and (**E**,**F**) the proposed dual-channel approach based on SpO_2_ and airflow jointly. AHI: apnoea-hypopnoea index; AHI_PSG_: actual AHI from polysomnography; SVM: support vector machine; SVM_SpO2_: regression SVM-based model for estimation of AHI from SpO_2_; SVM_AF_: regression SVM-based model for estimation of AHI from AF; SVM_SpO2+AF_: regression SVM-based model for estimation of AHI from joint analysis of SpO_2_ and AF.

The regression model SVM_SpO2_ trained with the optimum features from oximetry reached an ICC of 0.92 (95%CI 0.87–0.95) in the independent test dataset, whereas the SVM_AF_ model achieved 0.75 ICC (95%CI 0.62–0.85) using the selected features from airflow. The entire list of estimated AHI values from SVM_SpO2_ and SVM_AF_ models as well as the actual AHI values from at-home PSG can be found online as Supplementary Tables S3 and S4, respectively. The agreement between the estimated and the actual AHI was higher using the SVM_SpO2+AF_ model, which reached 0.93 ICC (95%CI 0.90–0.95). The estimated AHI values from the SVM_SpO2+AF_ dual-channel model can be found as Supplementary Table S5. Figure 3 shows the Bland-Altman and Mountain plots for qualitative assessment of the agreement between actual and estimated AHI.

**Figure 4:**
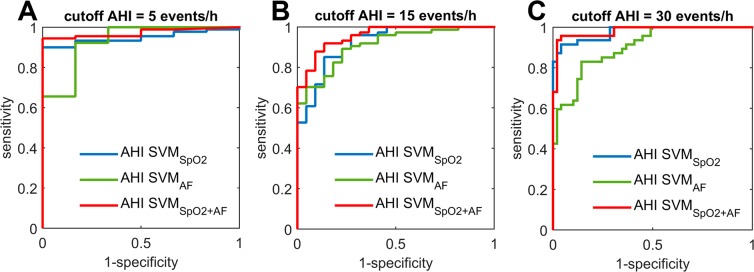
ROC curves for the AHI estimated using the proposed single-channel and dual-channel approaches using different cut-offs for positive OSA: (**A**) AHI = 5 events/h, (**B**) AHI = 15 events/h, and (**C**) AHI = 30 events/h. AHI: apnoea-hypopnoea index; SVM: support vector machine; SVM_SpO2_: regression SVM-based model for estimation of AHI from SpO_2_; SVM_AF_: regression SVM-based model for estimation of AHI from AF; SVM_SpO2+AF_: regression SVM-based model for estimation of AHI from joint analysis of SpO_2_ and AF.

Regarding the four common severity groups in the OSA context, 4-class kappa values equal to 0.61 (95%CI 0.46–0.75) and 0.42 (95%CI 0.25–0.58) were achieved using a single-channel approach based on oximetry and airflow, respectively, while a significantly higher (*p* < 0.01) agreement was reached using a dual-channel approach (0.71, 95%CI 0.58–0.84). Similarly, 4-class overall accuracy significantly increased (*p* < 0.01) from 75.0% (95%CI 64.3–84.6) for SVM_SpO2_ and from 61.5% (95%CI 49.8–72.1) for SVM_AF_ to 81.3% (95%CI 72.0–90.2) when using the optimum feature subset from SpO_2_ and airflow signals joint analysis. Table 3 shows the 4-class confusion matrices for the proposed approaches, whereas Tables 4–6 summarise the diagnostic assessment when setting a single fixed threshold for binary classification. Overall, SVM_SpO2+AF_ achieved the highest performance for the diagnosis of severe OSA (AHI ≥ 30 events/h), reaching 95.8% accuracy (95%CI 90.7–99.6) and 0.98 area under the ROC curve (AUC) (95%CI 0.95–1), as well as both sensitivity and specificity values above 90%. Figure 4 shows the ROC curves of each model for the three common cut-offs for OSA. Diagnostic performance maximises when using both SpO_2_ and airflow signals together, with AUC significantly higher (*p* < 0.01) than those achieved by SVM_SpO2_ and SVM_AF_, for all the cut-offs.

**Table 3:** Confusion matrices for a 4-class diagnostic assessment of the estimated AHI from automated pattern recognition of the proposed data sources.

		SVM_SpO2_				SVM_AF_				SVM_SpO2+AF_			
		NO OSA	MILD	MOD	SEV	NO OSA	MILD	MOD	SEV	NO OSA	MILD	MOD	SEV
PSG	NO OSA	**1**	4	1	0	**4**	1	1	0	**5**	1	0	0
	MILD	2	**5**	8	1	1	**9**	6	0	4	**6**	6	0
	MODERATE	0	2	**24**	1	1	6	**14**	6	0	3	**23**	1
	SEVERE	0	0	5	**42**	0	0	15	**32**	0	0	3	**44**

AF: airflow from nasal prong pressure; MILD: mild OSA; MOD: moderate OSA; OSA: obstructive sleep apnoea; SEV: severe OSA; SpO2: blood oxygen saturation from oximetry; SVM: support vector machine.

**Table 4:** Diagnostic assessment of the proposed models for estimation of the AHI using SpO_2_ and AF for a cut-off of 5 events/h for positive OSA in the independent test dataset.

Cut-off for positive OSA: AHI ≥ 5 events/h								
Approach	Se (%)	Sp (%)	PPV (%)	NPV (%)	LR +	LR−	Acc (%)	AUC
SVM_SpO2_	97.8 (93.9, 100)	16.7 (0.0, 84.4)	94.6 (88.7, 99.6)	33.3 (0.0, 100)	1.17 (0.94, 1.99)	0.13 (0.0, 0.26)	92.7 (86.1, 97.6)	0.95 (0.89, 1)
SVM_AF_	97.8 (93.8, 100)	66.7 (0.0, 100)	97.8 (93.6, 100)	66.7 (5.3, 100)	2.93 (0.98, 5.41)	0.03 (0.0, 0.12)	95.8 (90.6, 99.6)	0.93 (0.73, 1)
SVM_SpO2+AF_	95.6 (90.1, 99.6)	83.3 (18.4, 100)	98.9 (96.4, 100)	55.6 (7.4, 95.4)	5.73 (1.18, 6.29)	0.05 (0.0, 0.15)	94.8 (89.1, 99.6)	0.97 (0.92, 1)

AHI: apnoea-hypopnoea index; AF: airflow from nasal prong pressure; OSA: obstructive sleep apnoea; SpO2: blood oxygen saturation from oximetry; SVM: support vector machine.

**Table 5:** Diagnostic assessment of the proposed models for estimation of the AHI using SpO_2_ and AF for a cut-off of 15 events/h for positive OSA in the independent test dataset.

Cut-off for positive OSA: AHI ≥ 15 events/h								
Approach	Se (%)	Sp (%)	PPV (%)	NPV (%)	LR +	LR-	Acc (%)	AUC
SVM_SpO2_	97.3 (92.4, 100)	54.6 (28.1, 80.3)	87.8 (78.9, 95.7)	85.7 (55.3, 100)	2.14 (1.36, 5.11)	0.05 (0.0, 0.17)	87.5 (79.4, 94.3)	0.92 (0.84, 0.99)
SVM_AF_	90.5 (82.3, 98.8)	68.2 (42.7, 95.2)	90.5 (81.8, 98.7)	68.2 (41.3, 95.6)	2.85 (1.57, 7.54)	0.14 (0.02, 0.31)	85.4 (76.5, 93.3)	0.91 (0.83, 0.98)
SVM_SpO2+AF_	96.0 (90.0, 100)	72.7 (46.8, 96.7)	92.2 (84.2, 99.1)	84.2 (62.5, 100)	3.52 (1.84, 9.36)	0.06 (0.0, 0.15)	90.6 (83.1, 96.8)	0.96 (0.91, 1)

AHI: apnoea-hypopnoea index; AF: airflow from nasal prong pressure; OSA: obstructive sleep apnoea; SpO2: blood oxygen saturation from oximetry; SVM: support vector machine.

**Table 6:** Diagnostic assessment of the proposed models for estimation of the AHI using SpO_2_ and AF for a cut-off of 30 events/h for positive OSA in the independent test dataset.

Cut-off for positive OSA: AHI ≥ 30 events/h								
Approach	Se (%)	Sp (%)	PPV (%)	NPV (%)	LR +	LR-	Acc (%)	AUC
SVM_SpO2_	89.4 (78.3, 99.1)	95.9 (88.7, 100)	95.5 (87.4, 100)	90.4 (79.8, 99.2)	21.89 (7.52, 31.9)	0.11 (0.01, 0.23)	92.7 (86.2, 98.9)	0.98 (0.94, 1)
SVM_AF_	68.1 (51.5, 84.3)	87.8 (75.9, 98.6)	84.2 (69.2, 98.2)	74.1 (60.5, 87.5)	5.56 (2.71, 15.9)	0.36 (0.18, 0.57)	78.1 (67.8, 88.0)	0.90 (0.83, 0.97)
SVM_SpO2+AF_	93.6 (85.2, 100)	98.0 (93.0, 100)	97.8 (92.5, 100)	94.1 (85.3, 100)	45.9 (12.5, 34.8)	0.07 (0.0, 0.15)	95.8 (90.7, 99.6)	0.98 (0.95, 1)

AHI: apnoea-hypopnoea index; AF: airflow from nasal prong pressure; OSA: obstructive sleep apnoea; SpO_2_: blood oxygen saturation from oximetry; SVM: support vector machine.

## Discussion

In this study, we assessed the potential performance increase of simplified OSA screening tests when using both SpO_2_ and airflow recordings jointly. Signal processing and machine-learning methods were used to gain insight into the complementarity of these recordings in an unattended setting. A thorough automated feature selection procedure led to an optimum feature subset composed of variables from oximetry and airflow almost in the same proportion, which reinforces their joint relevance: 8 out of 18 (44.4%) derived from SpO_2_ and 10 out of 18 (55.6%) from airflow. Under a dual-channel approach, variables within the joint optimum feature subset were different compared with features selected in each single-channel approach, particularly airflow-derived variables (Fig. 2). While the histogram of relevance values for SpO_2_-derived features is very similar under both single- and dual-channel approaches, the profile for airflow-derived features is completely different. This suggests that airflow recordings contain essential information for OSA detection that is hidden when using the signal alone, while this complementary information arises when combined with overnight oximetry.

The estimated AHI from the optimum SVM_SpO2+AF_ model reached remarkable agreement with the actual AHI from PSG. Bland-Altman plots (Fig. 3) showed a small bias both using oximetry alone and using SpO_2_ and airflow jointly, with smaller dispersion under the dual-channel approach, particularly for AHI values <30 events/h. Overall limits of agreement were narrower when using oximetry and airflow together: confidence intervals of 32.45, 50.14, and 29.98 events/h were obtained using SpO_2_, airflow, and SpO_2_ + airflow, respectively. Accordingly, the performance of the dual-channel approach significantly outperformed individual SpO_2_ and airflow. AUC of SVM_SpO2+AF_ model was significantly higher (*p* < 0.01) for all diagnostic thresholds. Moreover, in contrast to single-channel approaches, balanced sensitivity-specificity pairs were always obtained. Concerning feasibility of out-of-centre portable devices to rule in OSA, Collop *et al*. established the criteria for ensuring appropriate accurateness^30^. Assuming a pre-test probability equal to the prevalence in our dataset for the different cut-offs, minimum LR+ values of 1.3, 5.6, and 19.8 would be needed to reach the recommended post-test probability of 95% in order to rule in mild, moderate, and severe OSA, respectively. The dual-channel approach notably outperformed these feasibility thresholds for mild (5.73, 95%CI 1.18–6.29) and severe (45.9, 95%CI 12.5–34.8) OSA, demonstrating the largest screening capability. In addition, the model simultaneously using both signals was the closest to the recommended limit for moderate-to-severe OSA.

According to the confusion matrix of the dual-channel SVM_SpO2+AF_ model shown in Table 3, the following screening protocol can be implemented in clinical practice: (i) if our model estimates an AHI < 5 events/h, then the physician could consider to follow-up patients and derive to PSG only if symptoms persists, since no moderate-to-severe OSA patients were categorised within the No OSA class and the 4 patients with mild OSA classified as No OSA actually had an AHI < 9 events/h; (ii) if our model estimates an AHI ≥ 30 events/h, then the physician could derive these patients for treatment, since 100% of subjects with an estimated AHI ≥ 30 events/h had at least moderate OSA with symptoms; (iii) patients with an estimated AHI between 5 and 30 will undergo PSG to confirm/discard the disease. Under this conservative protocol, 56.3% of PSGs (54 out of 96) would be potentially avoidable. Using a less conservative approach, with patients showing an estimated AHI ≥ 15 events/h directly referred for treatment since 100% of patients categorised as moderate-severe OSA had at least mild OSA with symptoms (71 out of 77 actually had moderate or severe OSA, while 6 out of 77 had mild OSA), the number of PSGs potentially avoidable would increase up to 89.6%.

To our knowledge, this is the first study that exhaustively analyses unattended SpO_2_ and airflow recordings jointly using machine-learning techniques. It is important to highlight two main novelties in this study. First, regarding healthcare resources, all the recordings were obtained at patient’s home, laying the foundations for an efficient simplified screening protocol able to decrease current overload of sleep laboratories. Previous studies highlight non-inferiority of at-home PSG in the management of OSA patients regarding both feasibility and repeatability, leading to shorter waiting times and substantial cost savings^31,32^. Nevertheless, simplified alternatives to complete PSG are needed to further decrease complexity and intrusiveness^33^. In this way, recent studies aimed at assessing abbreviated protocols at home against domiciliary PSG focus on single-channel approaches, mainly oximetry^25,34,35^. Chung *et al*. reported accuracies of 87.0%, 84.0%, and 93.7% for cut-offs of 5, 15, and 30 events/h, respectively^34^. Similarly, Gutiérrez-Tobal *et al*. reached accuracies of 92.9%, 87.4%, and 78.7% in the same thresholds^25^, whereas Schlotthauer *et al*. achieved 83.8% sensitivity and 85.5% specificity using a cut-off of 15 events/h^35^. In addition, several studies focused on the validation of single-channel airflow monitoring against in-laboratory PSG^36–40^. Poor performance and unbalanced sensitivity-specificity pairs were reported by Pang *et al*.^36^, while Rofail *et al*. reached 0.89 AUC for a cut-off of 5 events/h^38^. In the study by Oktay *et al*.^39^, sensitivity ranged from 55.6% to 76.9% and specificity from 76.9% to 95.5% for common diagnostic thresholds, whereas Crowley *et al*. reported sensitivity values ranging from 66.7% to 87.5% and specificities from 85.0% to 93.3%^40^. By contrast, Nakano *et al*. reported AUC values of 0.95, 0.96, and 0.98 for 5, 15, and 30 events/h using just a thermal sensor, although airflow and reference PSG were conducted in the hospital^37^.

A second novelty, from a machine-learning point of view, is that regression SVMs have been found to be high-performance tools able to accurately estimate the AHI using a reduced set of signals. Previous works already reached remarkable agreement between estimated AHI and PSG using both oximetry^23,41,42^ and airflow^16,17^ individually. Gutiérrez-Tobal *et al*. achieved 0.85 ICC using an artificial neural network fed with airflow-derived (thermistor) features^16^ and a 4-class kappa value of 0.43 applying ensemble learning to features from a nasal-prong pressure signal^17^. Using SpO_2_, Marcos *et al*. reached 0.91 ICC with a multivariate artificial neural network^23^ and Ebben & Krieger 0.88 ICC transforming the conventional ODI4 via quadratic regression analysis^41^. Furthermore, Jung *et al*. recently reported 0.99 ICC applying Hill regression to the ODI3^42^. Nevertheless, these studies were conducted in a hospital without prospective validation in unattended settings. On the other hand, the present study found that agreement and diagnostic performance might be improved using oximetry and airflow signals together.

Our proposal is a robust approach without significantly increasing the complexity and intrusiveness of portable monitoring. Indeed, commercial portable devices for simultaneous measurement of oximetry an airflow already exist, such as the widely known ARES and ApneaLink. Ayappa *et al*. and Masdeu *et al*. reported 0.80 ICC between in-lab PSG and semi-automated AHI from the ARES^43,44^. Similarly, Tonelli *et al*. reached AUC values of 0.96, 0.91, and 0.92 for cut-offs of 5, 15, and 30 events/h comparing manual AHI from ARES with in-lab PSG^45^. Using the ApneaLink, Gantner *et al*.^46^ and Chai-Coetzer *et al*.^47^ obtained sensitivity-specificity pairs of 86–85% and 88–82% in the detection of severe OSA compared to simultaneous PSG at home. Recently, Ward *et al*. reported sensitivities ranging from 43% to 80% and specificities ranging from 83% to 100% for the common cut-offs for OSA, although the reference PSG was conducted in the sleep laboratory in a separate night^48^.

Regarding the feasibility of unattended monitoring, in the present study 43 out of 299 (14.4%) at-home PSGs were discarded due to technical issues, mainly linked with EEG. Additionally, 6 (14.0%) PSGs were invalid due to low quality of airflow. Concerning the dual-channel approach, 17 out of 256 (6.6%) studies were removed after the pre-processing stage, of which 12 were invalid due to low quality of airflow. These numbers suggest that unsupervised airflow is more likely to be affected by artefacts than oximetry. In addition, beyond the valuable complementarity of both signals, our results revealed that the contribution of oximetry to the performance increase is greater than that of airflow. Therefore, the present study highlights again the importance of oximetry as a tool for simplified initial screening, especially to confirm severe OSA, where a PPV greater than 95% is reached, notably higher than single-channel airflow.

Some limitations should be considered. Despite the large at-home database used in the current study, more participants would increase the generalisability of our findings. In addition, although high OSA prevalence was observed in the sample, it agrees with the proportion of patients attended in sleep units. This is also consistent with the recommendations of the AASM regarding the use of portable abbreviated testing at home with patients showing high pre-test probability. Nevertheless, as machine-learning algorithms are known to be affected by unbalanced training datasets, this issue could influence our results.

Recent studies reported that the level of hypoxia is better correlated with mortality, cardiovascular disease or cancer incidence than conventional respiratory indexes based on the number of events per hour of sleep, such as the AHI or the ODI^49–51^. In this regard, novel estimates of hypoxia have been proposed, such as the hypoxic burden^51^, the hypoxia load^49^ or the desaturation severity parameter^52^. Our methodology includes different oximetry measures beyond the common indexes based on the number of desaturations, which could potentially account for this level of hypoxia, such as the frequency-domain (*M3f* and *PR*) and non-linear (*SampEn*, *CTM*, *LZC*) features included in the optimum model. Nevertheless, novel measures of hypoxia could increase the performance of the proposed methodology in the context of OSA screening. Concerning potential confounders that could influence our findings, the AASM recently demanded additional evidence on the effectiveness of abbreviated techniques for OSA screening in the presence of comorbidities, particularly cardiovascular and pulmonary diseases^6^. Therefore, further research is needed to confirm the accurateness of our dual-channel approach in patients with history of cardiovascular disease or suffering from COPD or obesity hypoventilation syndrome, among others.

## Conclusions

This study provides significant evidence on the superiority of a dual-channel approach in the framework of unattended abbreviated monitoring for OSA screening. Particularly, SpO_2_ and airflow signals have been found to provide complementary information leading to a remarkable performance increase compared to single-channel approaches. Our results also reveal that airflow recordings are more likely to be affected by permanent signal loss issues than oximetry in unattended settings. Nevertheless, we found that oximetry alone was able to maintain notably high accuracy, particularly in severe cases. We can conclude that joint analysis of simultaneous SpO_2_ and airflow recordings by means of machine-learning techniques provides accurate estimates of the AHI, which suggests its use as extensive routine screening test for OSA at home.

## Supplementary information

Supplementary Table S3.

Supplementary Table S4.

Supplementary Table S5.

Supplementary Information.

## Data Availability

All data generated during this study (estimated AHI) are included in this published article and its Supplementary Information Files. Additionally, the datasets (raw signals) analysed during the current study are available from the corresponding author on reasonable request.
