# Supplementary material for: A machine learning-based test for adult sleep apnoea screening at home using oximetry and airflow
Source: Sci Rep. 2020 Mar 24;10:5332. doi: 10.1038/s41598-020-62223-4 (PMC7093547; doi:10.1038/s41598-020-62223-4)
Supplement: Supplementary file 4 — Supplementary Information. [file 41598_2020_62223_MOESM4_ESM.docx]

**Supplementary material**

**A machine learning-based test for adult sleep apnoea screening at home using oximetry and airflow**

Daniel Álvarez, PhD,^1,2,3^ Ana Cerezo-Hernández, MD,^1^ Andrea Crespo, MD,^1,2^ Gonzalo C. Gutiérrez-Tobal, PhD,^2,3^ Fernando Vaquerizo-Villar, MSc,^2^ Verónica Barroso-García, MSc,^2^ Fernando Moreno, MSc,^1^ C. Ainhoa Arroyo, MD,^1^ Tomás Ruiz, MD,^1^ Roberto Hornero, PhD,^2,3^ Félix Del Campo, MD, PhD.^1,2,3^

**Authors’ Affiliations**

^1^Pneumology Department, Río Hortega University Hospital, Valladolid, Spain.

^2^Biomedical Engineering Group, University of Valladolid, Valladolid, Spain.

^3^Centro de Investigación Biomédica en Red en Bioingeniería, Biomateriales y Nanomedicina (CIBER-BBN), Spain.

**Corresponding Author**: Daniel Álvarez, Servicio de Neumología, Hospital Universitario Río Hortega, c/ Dulzaina 2, 47012, Valladolid, España. Telephone: +34 983420400 ext. 85776.

E-mail: [dalvarezgo@saludcastillayleon.es](mailto:dalvarezgo@saludcastillayleon.es)

**
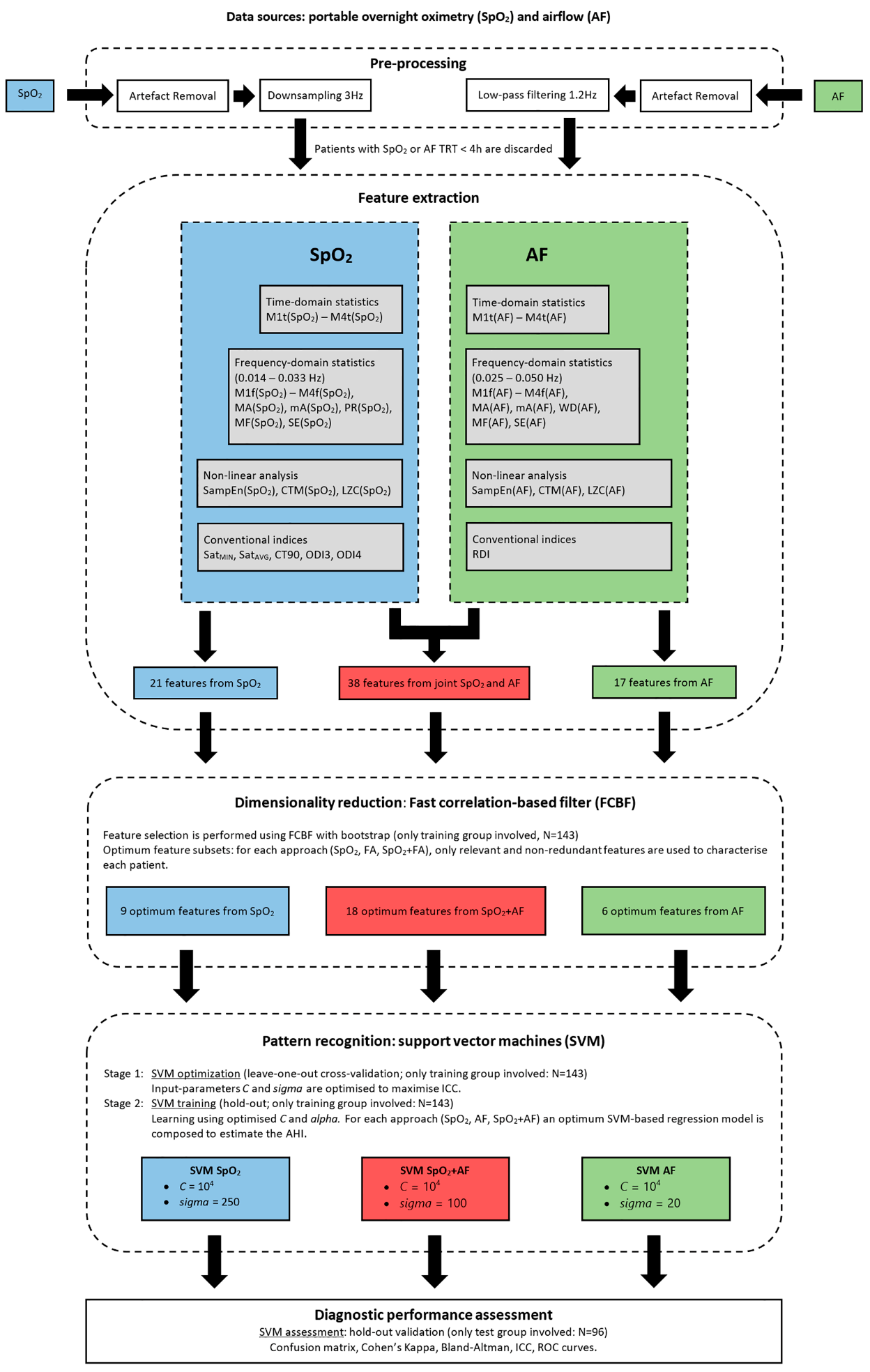
**

**Supplementary Fig. S1**. Flowchart showing the procedures and the datasets involved at each stage of the proposed signal-processing algorithm.

AF: airflow; SpO_2_: blood oxygen saturation; TRT: total recording time; M1t-M4t: 1^st^ to 4^th^ order statistical moments in the time domain; M1f-M4f: 1^st^ to 4^th^ order statistical moments in the apnoea-related frequency band; SE: Shannon spectral entropy; MF: median frequency; WD: Wootters distance; MA: maximum amplitude in the spectral band; mA: minimum amplitude in the spectral band; PR: relative power (*PR*); SampEn: sample entropy; CTM: central tendency measure; LZC: Lempel-Ziv complexity; ODI3: oxygen desaturation index of 3%; ODI4: oxygen desaturation index of 4%; Sat_MIN_: minimum saturation; Sat_AVG_: average saturation; CT90: cumulative time spent with a saturation below 90%; RDI: respiratory disturbance index; FCBF: fast correlation-based filter; SVM: support vector machine; SVM_SpO2_: regression SVM-based model for estimation of AHI from SpO_2_; SVM_AF_: regression SVM-based model for estimation of AHI from AF; SVM_SpO2+AF_: regression SVM-based model for estimation of AHI from joint analysis of SpO_2_ and AF; ICC: intra-class correlation coefficient.

**
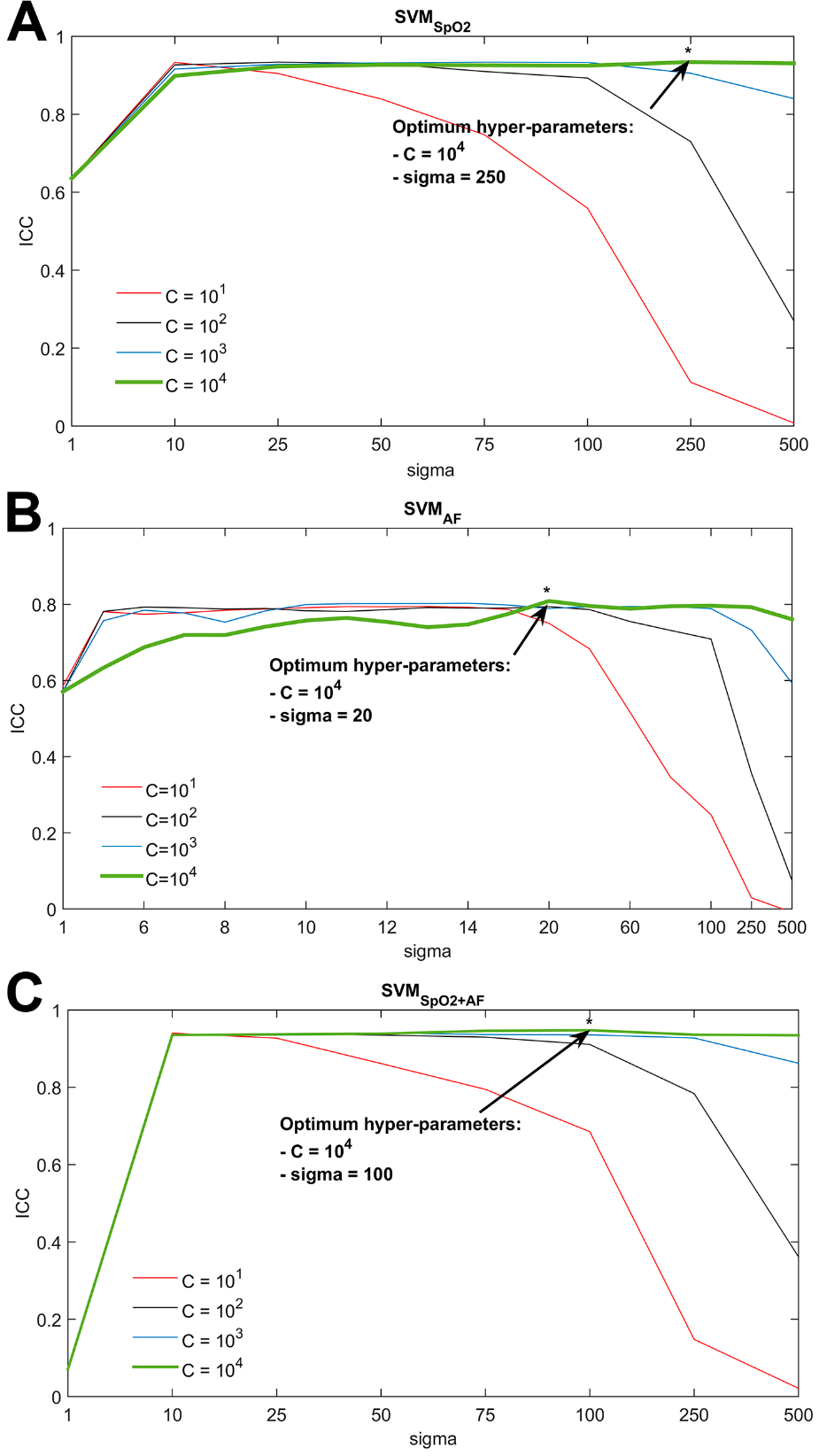
**

**Supplementary Fig. S2**. Optimisation of the SVM input-parameters *C* (regularisation) and *sigma* (Gaussian kernel width) for each model: (A) single-channel SpO_2_, (B) single-channel airflow, and (C) the proposed dual-channel approach based on SpO_2_ and airflow jointly. The optimum (*C*, *sigma*) pair is determined as the point where ICC maximises.

AF: airflow; SpO_2_: blood oxygen saturation; SVM_SpO2_: regression SVM-based model for estimation of AHI from SpO_2_; SVM_AF_: regression SVM-based model for estimation of AHI from AF; SVM_SpO2+AF_: regression SVM-based model for estimation of AHI from joint analysis of SpO_2_ and AF; ICC: intra-class correlation coefficient.

**Supplementary Table S3**. Estimated AHI values from the SVM_SpO2_ single-channel model as well as the actual AHI from at-home PSG in the test dataset.

**Supplementary Table S4**. Estimated AHI values from the SVM_FA_ single-channel model as well as the actual AHI from at-home PSG in the test dataset.

**Supplementary Table S5**. Estimated AHI values from the SVM_SpO2+FA_ dual-channel model as well as the actual AHI from at-home PSG in the test dataset.
